# Potential using of infrared thermal imaging to detect volatile compounds released from decayed grapes

**DOI:** 10.1371/journal.pone.0180649

**Published:** 2017-06-30

**Authors:** Luyu Ding, Daming Dong, Leizi Jiao, Wengang Zheng

**Affiliations:** National Engineering Research Center for Information Technology in Agriculture, Beijing Academy of Agriculture and Forestry Sciences, Beijing, China; UC Davis MIND Institute, UNITED STATES

## Abstract

Previous studies have demonstrated variations in volatile compound content during fruit spoilage. Infrared spectroscopy was proposed as an alternative method to discriminate the various states of decayed fruit through the makeup of their volatile compounds. Based on the infrared spectra of volatile compounds obtained from decayed grapes, this study simplified the extraction of their feature spectra and visualized their gas plumes by using a commercial infrared thermal camera equipped with a custom-made wavelength filter. As a function of volatilization gradients, accumulated gray value and imaging area were proposed as indicators for semi-quantitative analysis in a volatilization range similar to that of ethanol solutions ranging from 10% to 70%. Fresh, seriously decayed, and slightly or moderately decayed grapes were rapidly discriminated through their alcoholic volatiles by thermal images with correct classification ratings of 100%, 93.3%, and 90%, respectively.

## Introduction

Table grapes deteriorate rapidly during postharvest storage. Loss of quality accompanied with berry decay of table grapes during storage reduces their shelf-life and compromises food safety in marketing [1]. Thus, a simple and rapid way to detect decayed grapes is essential for quality control and to minimize economic loss.

In general, fruits contain specific volatile compounds, and the types or ratios of the compounds vary during berry development and deterioration [2–4]. Thus, the specific type of volatile compounds from fruit was proposed as a potential indicator for rapid detection of spoilage [5–7]. To date, gas chromatography-mass spectrometer (GC-MS) has been the most widely adopted method for precision analysis of volatile compounds from fruits [8–10]. More recently, other methods such as electronic nose (E-nose) and infrared spectroscopy have been developed as alternative means for quick analysis and to assess fruit freshness with high sensitivity [4, 10, 11]. However, for GC-MS and E-nose analysis, both methods require gas sampling prior to quantitative analysis; this complicates the measurement procedure and restricts their use in mass storage. In our previous studies, we successfully used infrared spectroscopy for gas measurements during grape or strawberry storage and food fermentation. This technique has the significant advantages of being able to provide nonintrusive, rapid and continuous online monitoring [4, 10, 12, 13]. Feature spectra of alcohols and esters were achieved and applied to discriminate grape spoilage [10]. However, gases had to be sampled and pumped into a cell equipped with multiple reflection mirrors to increase the optical paths because of the measurement limitations of infrared spectroscopy.

In recent years, gas plume visualization has been achieved by using an imager paired with a filter based on the specific wavelength band of the gas of interest [14–17]. This makes simple, rapid gas monitoring possible through the acquisition of infrared thermal images and lowers the determination costs related to infrared spectroscopy. This study examined the use of infrared thermal imaging using a wavelength filter to assess the freshness of table grapes based on their volatile compounds. Before applying the method to the assessment of grape freshness, preliminary tests were conducted to detect the vapors from ethanol solutions in different concentration gradients.

## Material and methods

### Experimental set-up

**Fig 1:**
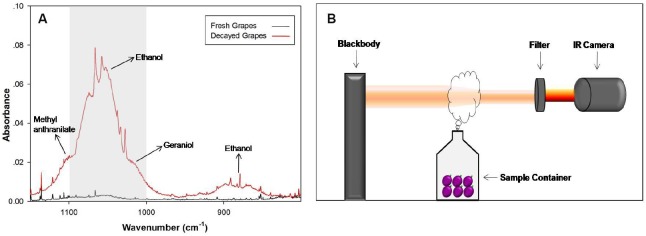
Schematic diagram of the experimental set-up and the wavelength band chosen for the filter. (A) Infrared feature spectra of alcohols released from decayed grapes, as recorded in our previous study. The marked area shows the wavelength band chosen in this study (redrawn from Dong et al. [10]). (B) Non-dimensional diagram of the experimental set-up.

Fig 1B shows the experimental set-up used in this study. The main components include a standard blackbody, a sample container, a wavelength filter, and a commercial infrared thermal camera (IR camera). The standard blackbody (ISO-TECH R982, UK) operated at 78°C to serve as a constant imaging background. It was connected to a power supply (220V) and used its internal resistance for temperature control. Sample containers were placed in front of the blackbody to obtain thermal images before and after the sample container caps were removed. Thermal images were obtained by the IR camera (FLIR SC620, USA; resolution of 480×640 pixels) located opposite the blackbody. Prior to taking images, a radiance of 1.0 and a reflected background temperature of 78°C at a distance of 0.3 m were set as the target parameters for the IR camera. The lens of the IR camera was equipped with a custom-made wavelength filter (BP-9480-613, Spectrogon, Sweden) that permitted transmission in the range of 1000–1100 cm^-1^. Fig 1A shows the infrared spectra of volatile compounds released from decayed grapes as recorded in our previous study [10]. The wavelength band of the filter was chosen based on the feature spectra of the alcohols (marked as gray in Fig 1A) released from decayed grapes.

### Solution preparation

A series of nine ethanol solutions (10–90% v/v) were prepared by dilution of 75% or 95% ethanol solution with deionized water. A 100-ml portion of each diluted ethanol solution was transferred into a 150-ml transparent plastic container for measurements.

### Grape preparation and classification

Experiments in this study used the *Jufeng* grape cultivar. It is commercially available and was obtained from a fruit chain store (Guo Xiang Si Ye) located close to the Beijing Academy of Agriculture and Forestry Sciences (BAAFS), Beijing, China. Fresh grape berries (108.6±6.0 g) with short stems (about 0.5 cm) were stored in 150-ml plastic containers (as used for ethanol solutions) at 18°C for days to reach different spoilage stages classified as: 1) fresh (FR), stored for 1–3 days; 2) slightly decayed (SL), stored for 4–6 days, visually intact except for stem browning; 3) moderately decayed (MO), stored for 7–9 days, visually softened, some with mildew stains at stem joints; 4) seriously decayed (SR), stored for 10–12 days, berries were visually juicy and some were cracked. Normally, grapes without any treatment will be seriously decayed within 7 days at room temperature [10]. According to information obtained as part of our investigation, commercial grapes are immersed in and cleaned with Kemeiling (a mold inhibitor) and then fumigated with fresh-keeping agents for 20 min before packaging. Because of the preservative treatment of commercial grapes, the duration of storage required to reach seriously decayed condition was lengthened in this study.

**Fig 2:**
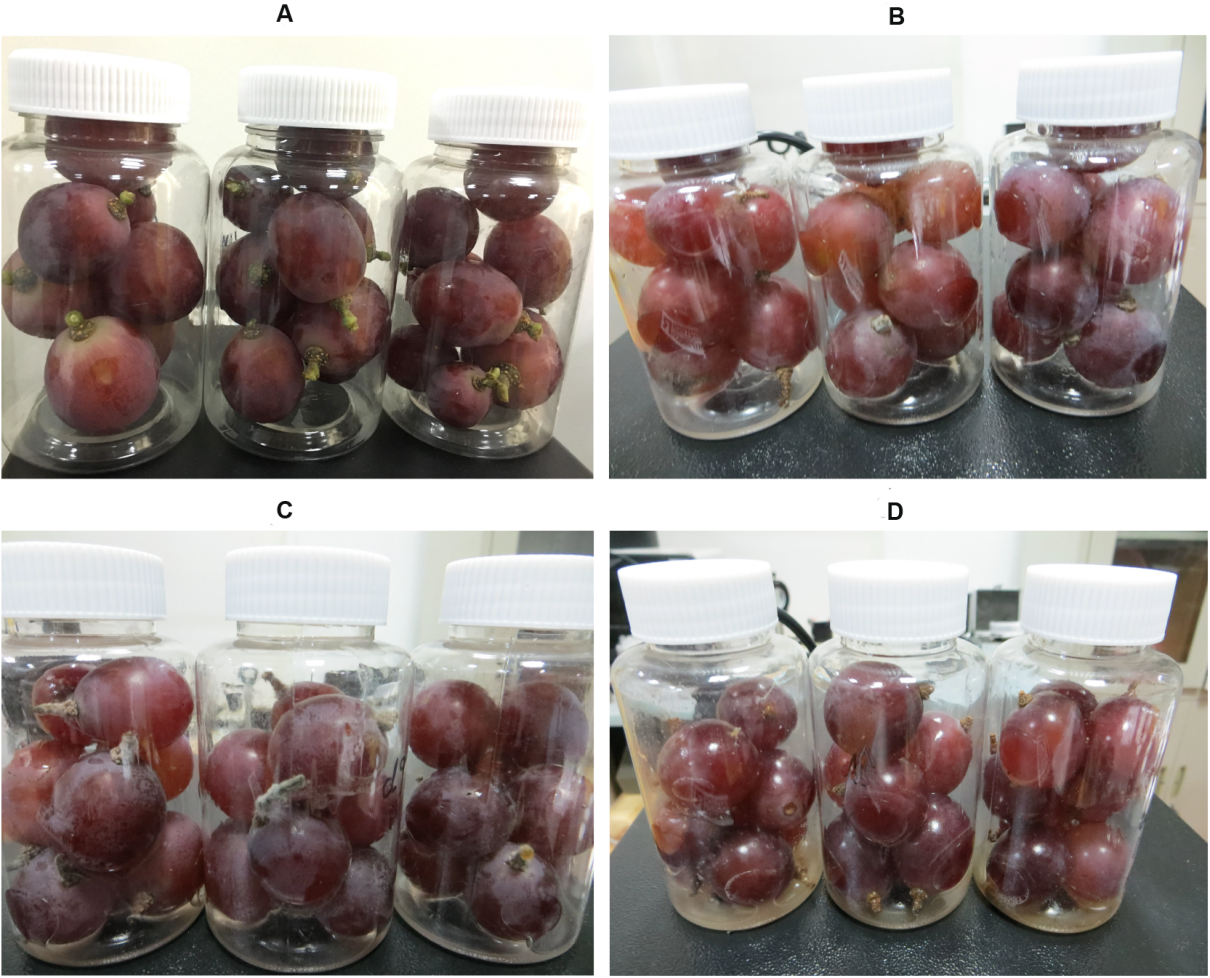
Grape samples at different spoilage stages. (A) Fresh, (B) slightly decayed, (C) moderately decayed, and (D) seriously decayed.

Images of the grape samples at different spoilage stages are shown in Fig 2. During storage, the plastic containers were not sealed (caps off) to allow normal air exchange with the atmosphere. The humidity of the air during storage was around 40%.

### Image acquisition and processing

Vapors from ethanol solutions were monitored by an IR camera as a feasibility analysis prior to the measurements of table grapes. Sample containers containing ethanol solutions or grape berries were placed in front of the blackbody to obtain thermal images before and after the container caps were removed. Prior to the recording of images, samples were shaken (caps on) to improve gas diffusion. The measurement of each spoilage stage of grapes used 15 replicates, while each concentration of ethanol solutions used 5 replicates. During the measurements, the air temperature was 19.8 ± 1.8°C and the humidity was 39 ± 5%.

Thermal images taken before and after the container caps were removed (cap-on and cap-off) were adjusted to the same background in a temperature span of 2°C by FLIR QuickReport (version 1.2) software. Because of the absorption of cold gases, radiation from the blackbody was expected to be attenuated when the caps were removed [18]. Thus, visualization of gas plumes was achieved by subtracting cap-off images from the cap-on images in grayscale through Matlab software (version 7.0). To enhance the imaging signals, images of gas plumes were amplified five times. Histogram equalization and a 3×3 median filter was applied to the amplified images to remove signal noise. To demonstrate gaseous volatilization from sample containers more vividly, gas plume images were overlaid on the corresponding cap-on images and displayed in false color.

### Statistical analysis

Considering the central 160 × 190 region in the gas plume images, the accumulated gray value (AGV) and the actual imaging area (AIA) in the region were defined and calculated for data analyzing. AGV was defined as the sum of the gray value in each pixel before image amplification. AIA was defined as the total number of pixels with a gray value over zero. Based on AGV and AIA in the gas plume images, analysis of variance (ANOVA) and *T*-test were used for statistical analysis by the Matlab software (version 7.0). Classification of grapes in different spoilage stages was performed by Euler Clustering using Unscrambler software (version 9.7). Error bars in this study represent the standard errors.

## Results and discussion

### Ethanol vapors monitored by infrared thermal imaging

Alcohols make up a large proportion in the volatile compounds released from decayed grapes [10, 19] and most of them can be characterized in the wavelength band of infrared spectroscopy used in this study (Fig 1A). Hence, initial trials monitored vapors from ethanol solutions of known concentration using the experimental set-up.

**Fig 3:**
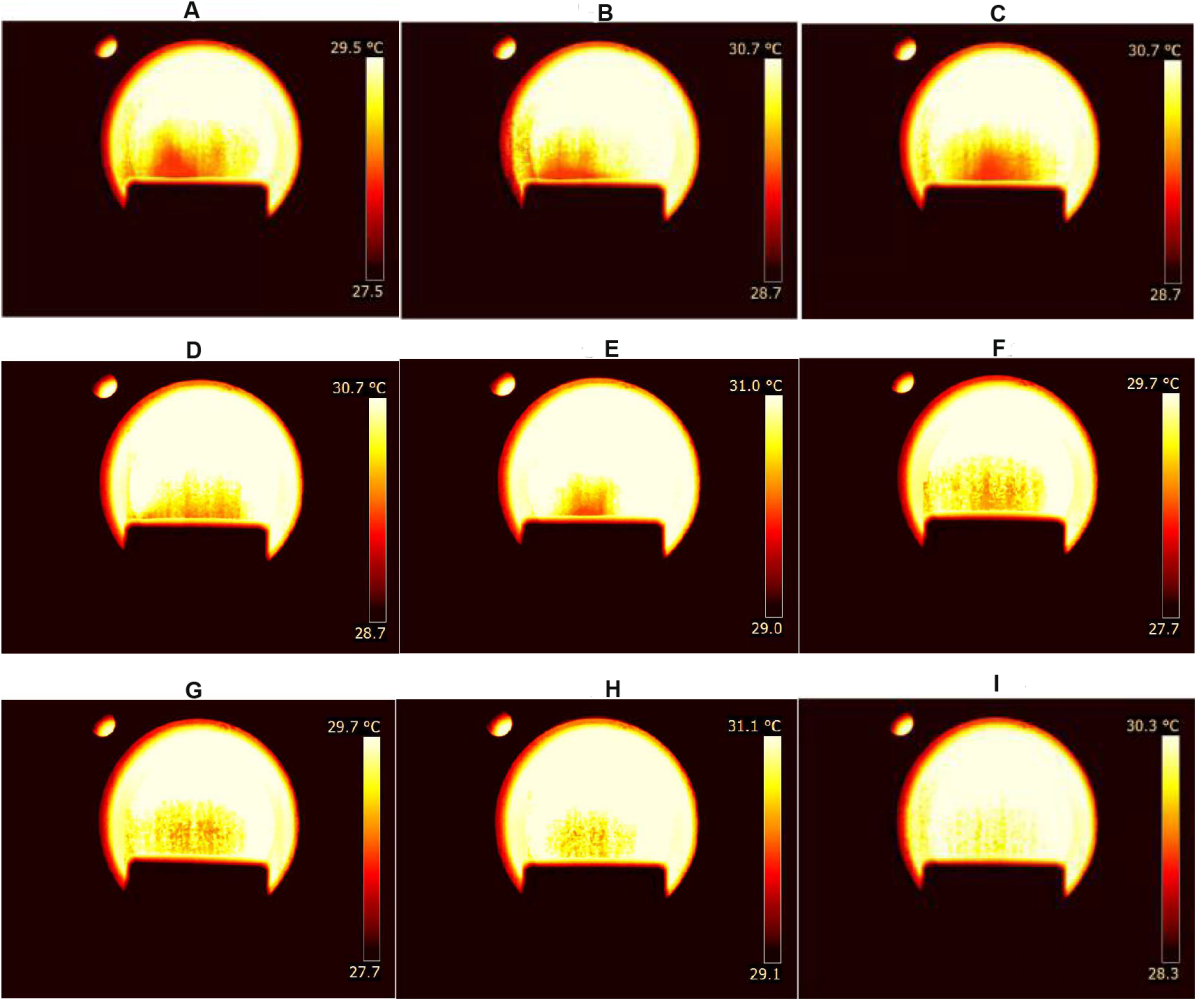
Images of vapors released from ethanol solutions of different concentrations. An example of false color images of visualized gas plumes when the ethanol concentration ranges from 90% to 10% (from A to I).

False color images of the ethanol vapors released from the different solutions are shown in Fig 3. By using the wavelength filter, gas plumes could be visualized even though the ethanol concentration was as low as 10% (Fig 3I). According to our unpublished data, gas plumes could be visualized only when the ethanol concentration was higher than 50% if no filter was used. In addition, the imaging area without a filter was quite small, which made the images of gas plumes rather vague and difficult to recognized. Therefore, we consider that use of a wavelength filter greatly enhance the quality and sensitivity of infrared thermal imaging in monitoring gaseous volatilization.

**Fig 4:**
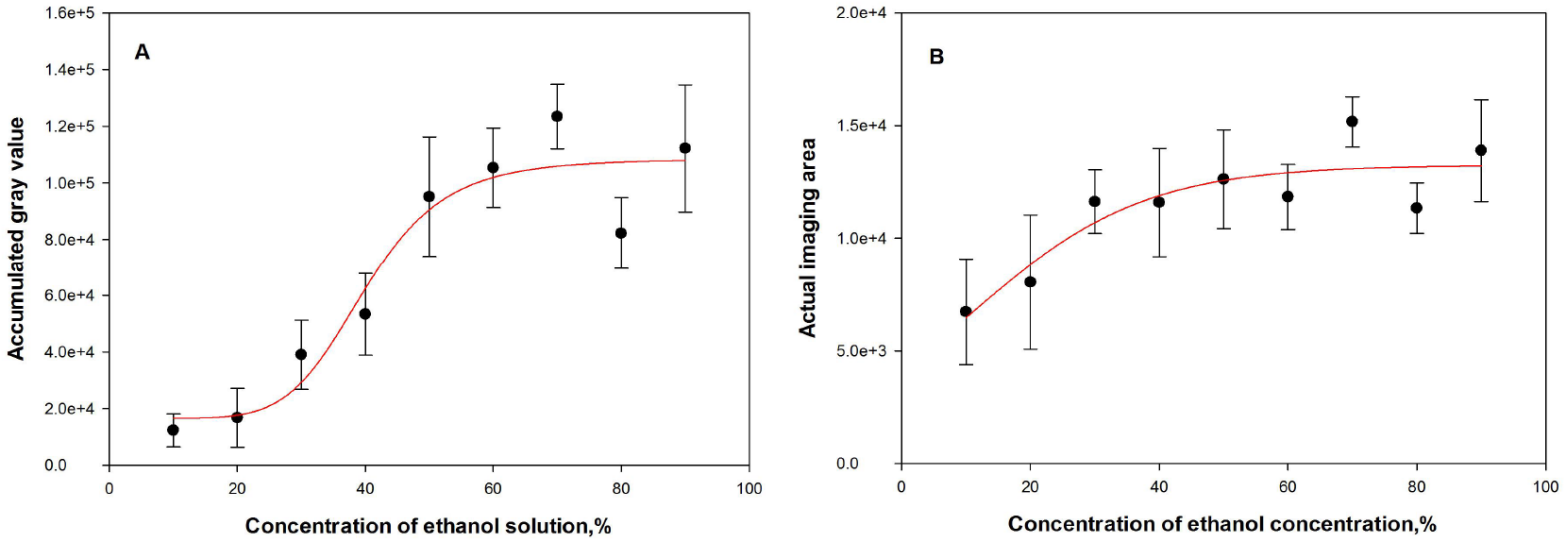
Averaged AGV and AIA of vapor images of volatiles released from ethanol solutions of different concentrations. The accumulated gray value (A) and actual imaging area (B) obtained from the images of gas plumes prior to being overlaid on the corresponding cap-on images when ethanol solution concentration ranged from 90% to 10% (error bars represent the standard errors, similarly hereinafter).

Fig 4A shows the averaged AGVs of gas plume images obtained from the series of ethanol solutions. Based on the results of ANOVA, the concentration gradient had a significant impact on the AGV in the images (*P* < 0.05).

According to Raoult's law [20, 21], a higher solution concentration gives rise to a higher saturated vapor pressure. This makes the solution more volatile at a higher concentration. In general, the AGV of the gas image was positively related to the concentration of ethanol solution (Fig 4A). This relationship is analogous to the trend described by Raoult's law, and suggests that the AGV in infrared thermal images could be employed as a semi-quantitative indicator within a specific volatilization range. However, measured AGVs tended to be saturated when the ethanol concentration was over 70% or under 30% (Fig 4A) on the basis of curve fitting (*P* < 0.05). This indicates that the effective detection range of thermal imaging used in this study may cover the volatilization range of ethanol solutions from 30% to 70%.

Curve fitting of averaged AIA in gas plume images is shown in Fig 4. Differing from AGV, AIA decreased rapidly for ethanol concentrations below 40%, but remain relatively stable for concentrations above 40% (Fig 4B). Thus, taking AIA as an additional indication, semi-quantitative analysis of gaseous volatilization through infrared thermal images can be extended to lower concentrations to give a combined analytical scale for ethanol solutions ranging from 10% to 70%.

In summary, alcohol vapors, particularly that of the ethanol, can be effectively monitored and semi-quantitatively analyzed by thermal imaging. Base on AGVs and AIAs in the images of gas plumes, the upper and lower detecting limitations were similar to the volatilization of ethanol solutions ranging from 70% down to 10%.

### Volatile compounds from decayed grapes monitored by infrared thermal imaging

After acquiring gas plume images from ethanol solution, volatile compounds from grapes at different spoilage stages were monitored by the same experimental set-up. Data obtained from the gas plumes of ethanol solutions provided crucial criteria for monitoring and analyzing the volatiles from grapes by thermal imaging.

**Fig 5:**
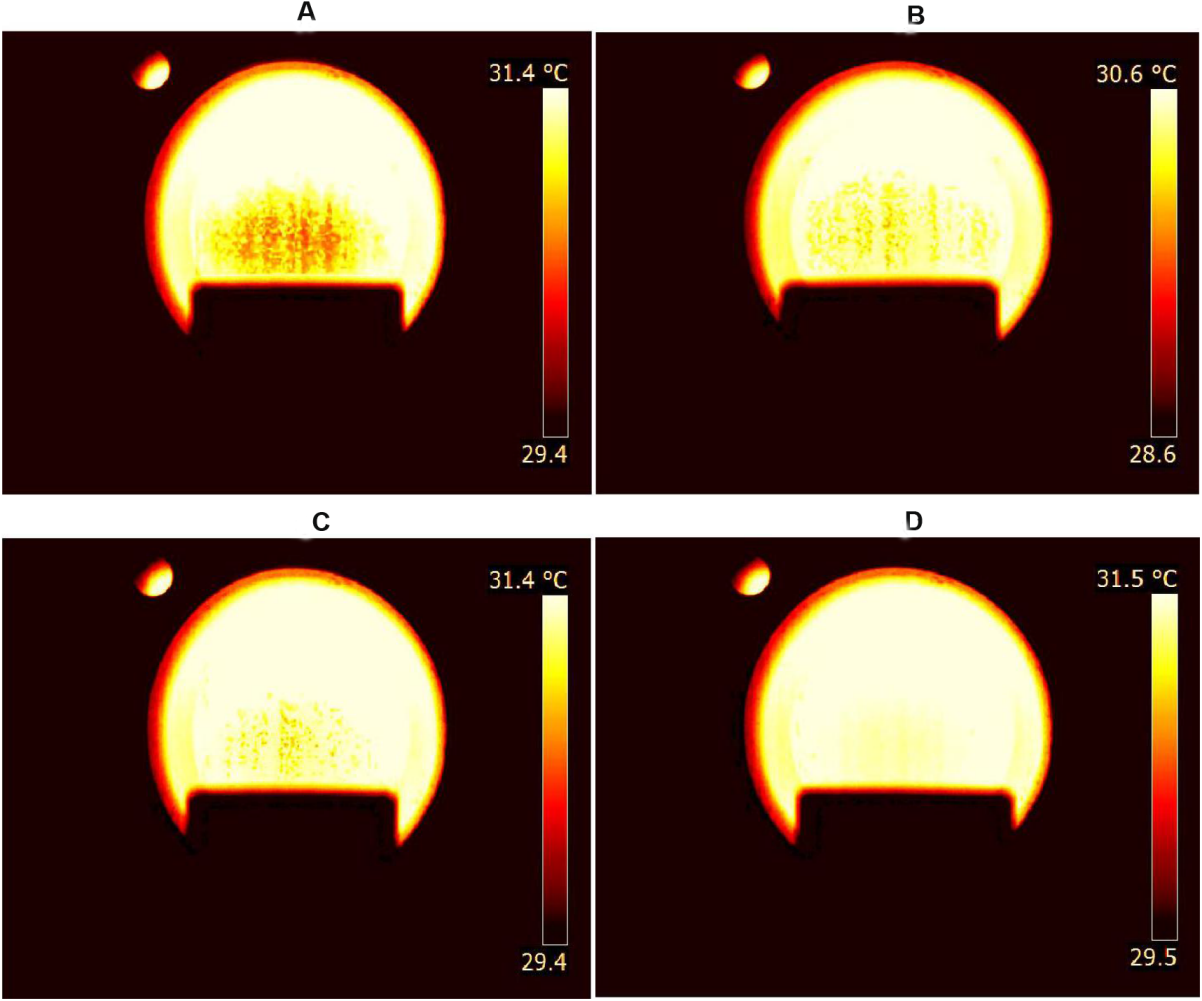
Images of volatile compounds released during grape spoilage. False color images of visualized gas plumes from grapes that were seriously decayed (A), moderately decayed (B), slightly decayed (C) and fresh (D).

Fig 5 shows an example of false color images of volatile compounds during grape spoilage. Gas plumes from decayed grapes can be clearly seen (Fig 5A–5C), while that from refresh grapes is barely visible (Fig 5D). In addition, the brightest image and the largest imaging area were observed when the grapes were seriously decayed. This is consistent with the results obtain by Fourier-transform-infrared spectroscopy (FTIR) [10], which showed higher ethanol evaporation from grapes during longer storage. Based on our unpublished results, gas plumes were very difficult to visualize without using the filter even when the grapes were seriously decayed. Hence, infrared thermal imaging through a wavelength filter is an effective way to monitor volatile compounds from grapes during spoilage.

**Fig 6:**
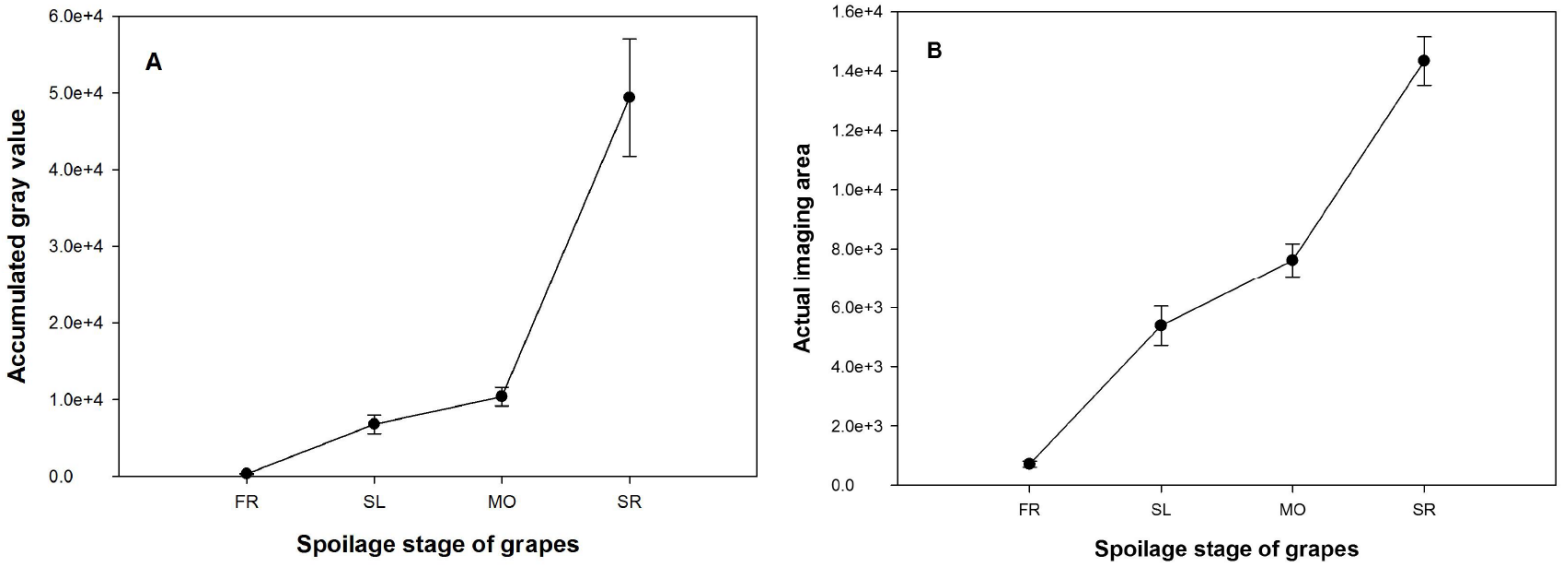
Averaged AGV and AIA of thermal images during grape spoilage. The accumulated gray value (A) and actual imaging area (B) obtained from the images of gas plumes prior to being overlaid on the corresponding cap-on images when grapes were fresh (FR), slightly decayed (SL), moderately decayed (MO), or seriously decayed (SR).

Fig 6A shows the averaged AGV of imaged grape volatiles. Notably, AGV positively related to grape spoilage, as did AIA (Fig 6). Fresh grapes had a significantly lower AGV (*P* < 0.05), which was less than 1.0 × 10^3^ (Fig 6A). When the grape was slightly or moderately decayed, gas plumes were visible in the images (Fig 5B and 5C) and the averaged AGV or AIA was close to that of the vapors released from ethanol solutions in the concentration range of 10–20%. In this range, the AGV was generally less than 2.0 × 10^4^ (Figs 4 and 6). However, statistics analysis did not show a significant difference of AGV between fresh and slightly decayed grapes or between slightly and moderately decayed grapes because of their large variances (*P* > 0.05). When compared with slightly or moderately decayed grapes, seriously decayed grapes had a much higher AGV (*P* < 0.05). The averaged AGV for seriously decayed grapes was 4.9 × 10^4^, which is close to that observed for 30% and 40% ethanol solution (Figs 4A and 6A).

AGVs in the thermal images of volatile from grapes were close to those from ethanol solutions under 40%, suggesting that AIA may be essential information for images from different spoilage stages. Statistics analysis showed similar results between AIA and AGV. This suggests that infrared thermal imaging may have difficulty in discriminating between slightly decayed and moderately grapes.

### Identification of grape spoilage through infrared thermal imaging

**Fig 7:**
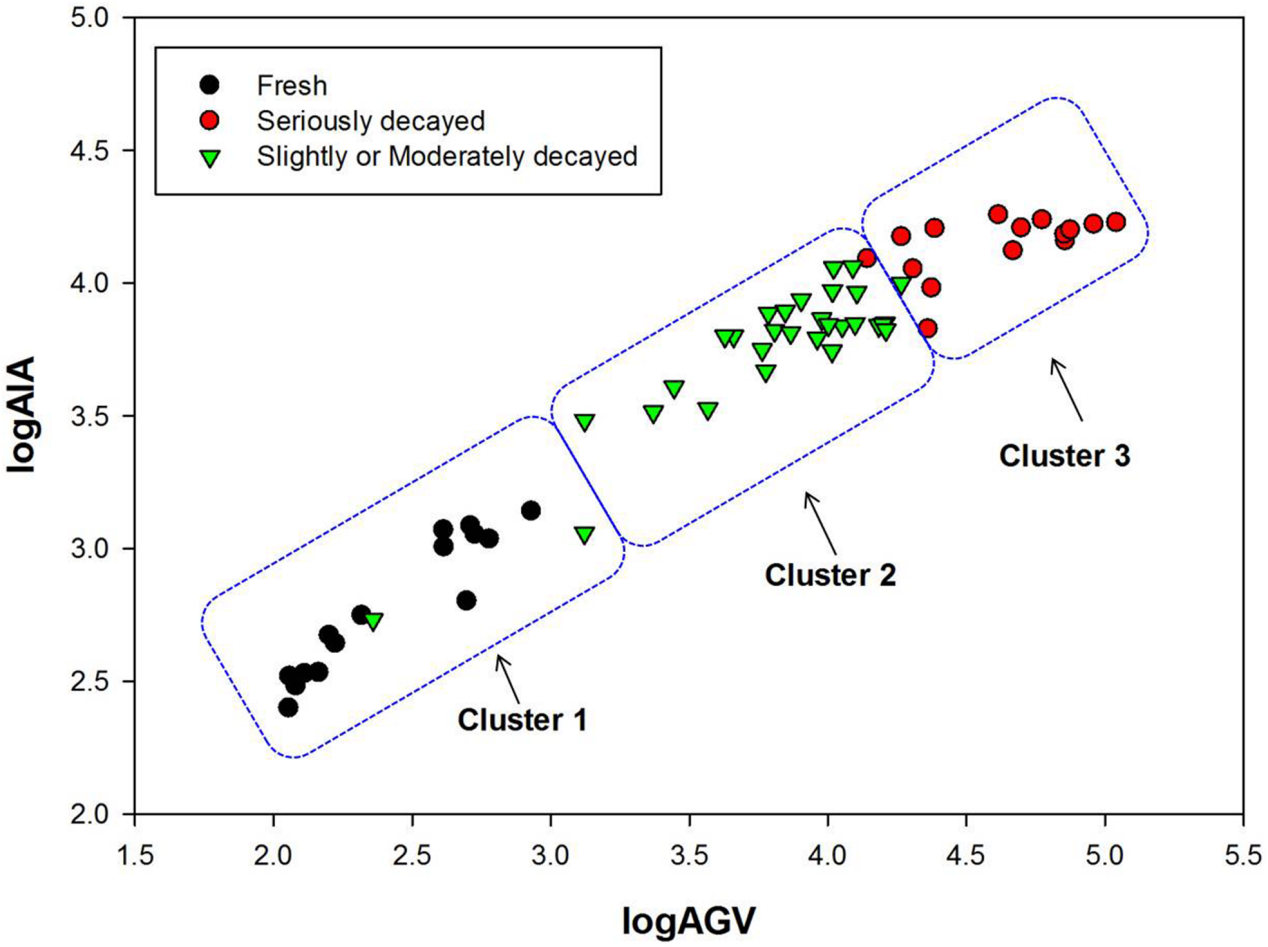
Clustering of grapes during spoilage. Grapes were classified into three clusters through Euclidean distance by taking the logarithm of AGV and AIA in the thermal images of volatile compounds released from different spoilage stages of grapes.

Based on the release of volatile compounds from grapes, Euler Clustering was applied to the logarithm of AGV and AIA in their thermal images to classify grapes into different spoilage stages. Grapes were classified into three clusters according to the statistical analysis (Fig 7). Table 1 shows the results of clustering to discriminate grapes during spoilage. Fresh grapes were accurately identified with a 100% correct classification (Fig 7, Table 1). Mistakes occurred when identifying the decayed grapes. Slightly or moderately decayed grapes were most likely to be misidentified and had a 6.7% error rate to be clustered as fresh grapes or a 3.3% error rate to be clustered as seriously decayed grapes (Fig 7, Table 1). Despite the misidentification of decayed grapes, the correct classification of grape spoilage was generally over 90%. Hence, fresh, seriously decayed, as well as slightly and moderately decayed grapes can be identified simply and quickly through their volatile compounds by infrared thermal imaging with a specific wavelength filter.

**Table 1:** Results of clustering to identify the spoilage stage of grapes.

Category	Sample size	Clustered as FR	Clustered as SL&MO	Clustered as SR	Error, %
FR	15	15	0	0	0.0
SL&MO	30	2	27	1	10.0
SR	15	0	1	14	6.7

## Conclusions

Infrared thermal imaging through a specific wavelength filter was developed as a rapid, flexible, nonintrusive method to monitor volatile compounds directly from decayed table grapes. Using a wavelength filter of 1000–1100 cm^-1^, gas plumes from decayed grapes were monitored by thermal imaging with relatively high sensitivity with similar volatilization shown by ethanol solutions in the range of 10–70%. The results suggest that the method could be used to discriminate the spoilage of fruit or other foods based on their volatile compounds. Based on the AGVs and AIAs in the gas plume images, slightly decayed or seriously decayed grapes were rapidly discriminated from fresh grapes, but moderately decayed grapes were difficult to distinguish from slightly decayed grapes.

## Supporting information

S1 Table Raw data of the sum of the gray value in each pixel in the images of ethanol vapors collected from different concentrations of ethanol solution by infrared thermal imaging. (DOC)

S2 Table Raw data of the total number of pixels with a gray value over zero in the images of ethanol vapors collected from different concentration of ethanol solutions by infrared thermal imaging. (DOC)

S3 Table Raw data of accumulated gray values (AGV) and actual imaging area (AIA) in the images of volatile compounds from grapes in different spoilage stages by infrared thermal imaging. FR, SL, MO and SR in the table represent fresh grapes, slightly decayed grapes, moderately decayed grapes and seriously decayed grapes, respectively.
(DOC)
