# Supplementary material for: Potential using of infrared thermal imaging to detect volatile compounds released from decayed grapes
Source: PLoS One. 2017 Jun 30;12(6):e0180649. doi: 10.1371/journal.pone.0180649 (PMC5493428; doi:10.1371/journal.pone.0180649)
Supplement: S1 Table — (DOC) [file pone.0180649.s001.doc]

**S1 Table. Raw data of the sum of the gray value in each pixel in the images of ethanol vapors collected from different concentrations of ethanol solution by infrared thermal imaging.**

| Concentration | 90% | 80% | 70% | 60% | 50% | 40% | 30% | 20% | 10% |
| --- | --- | --- | --- | --- | --- | --- | --- | --- | --- |
| Replicate 1 | 1,399,616 | 830,314 | 1,831,048 | 1,183,425 | 561,115 | 564,395 | 516,146 | 26,579 | 1,136 |
| Replicate 2 | 502,917 | 829,405 | 1,291,785 | 810,593 | 1,271,937 | 247,561 | 774,897 | 2,831 | 83,465 |
| Replicate 3 | 1,805,447 | 805,967 | 1,409,532 | 631,195 | 729,940 | 821,665 | 90,460 | 40,962 | 253,581 |
| Replicate 4 | 1,055,424 | 858,536 | 1,377,756 | 1,370,697 | 572,445 | 160,308 | 176,991 | 209,984 | 7,732 |
| Replicate 5 | 845,478 | 861,954 | 858,750 | 1,027,844 | 1,620,232 | 872,048 | 391,399 | 556,817 | 266,743 |
| Mean | 1,121,776 | 837,235 | 1,353,774 | 1,004,751 | 951,134 | 533,195 | 389,979 | 167,435 | 122,531 |
| SE | 224,536 | 103,72 | 154,978 | 131,075 | 211,548 | 144,852 | 122,302 | 1,040,09 | 58,057 |
