# Supplementary material for: Potential using of infrared thermal imaging to detect volatile compounds released from decayed grapes
Source: PLoS One. 2017 Jun 30;12(6):e0180649. doi: 10.1371/journal.pone.0180649 (PMC5493428; doi:10.1371/journal.pone.0180649)
Supplement: S2 Table — (DOC) [file pone.0180649.s002.doc]

**S2 Table. Raw data of the total number of pixels with a gray value over zero in the images of ethanol vapors collected from different concentration of ethanol solutions by infrared thermal imaging.**

| Concentration | 90% | 80% | 70% | 60% | 50% | 40% | 30% | 20% | 10% |
| --- | --- | --- | --- | --- | --- | --- | --- | --- | --- |
| Replicate 1 | 16,106 | 14,877 | 21,065 | 16,989 | 9,108 | 16,551 | 15,228 | 5,258 | 329 |
| Replicate 2 | 7,795 | 9,908 | 17,957 | 8,247 | 19,175 | 7,812 | 14,503 | 717 | 8,954 |
| Replicate 3 | 20,814 | 11,282 | 16,443 | 10,445 | 11,924 | 15,674 | 8,340 | 6,004 | 10,485 |
| Replicate 4 | 14,262 | 9,270 | 12,436 | 12,094 | 7,099 | 4,160 | 8,813 | 9,788 | 1,976 |
| Replicate 5 | 10,467 | 5,348 | 13,837 | 11,393 | 15,797 | 13,720 | 11,217 | 18,475 | 11,907 |
| Mean | 13,889 | 10,137 | 16,348 | 11,834 | 12,621 | 11,583 | 11,620 | 8,048 | 6,730 |
| SE | 2,257 | 1,541 | 1,524 | 1,443 | 2,195 | 2,401 | 1,416 | 2,979 | 2,339 |
