# Supplementary material for: Potential using of infrared thermal imaging to detect volatile compounds released from decayed grapes
Source: PLoS One. 2017 Jun 30;12(6):e0180649. doi: 10.1371/journal.pone.0180649 (PMC5493428; doi:10.1371/journal.pone.0180649)
Supplement: S3 Table — FR, SL, MO and SR in the table represent fresh grapes, slightly decayed grapes, moderately decayed grapes and seriously decayed grapes, respectively. (DOC) [file pone.0180649.s003.doc]

**S3 Table. Raw data of accumulated gray values (AGV) and actual imaging area (AIA) in the images of volatile compounds from grapes in different spoilage stages by infrared thermal imaging.** FR, SL, MO and SR in the table represent fresh grapes, slightly decayed grapes, moderately decayed grapes and seriously decayed grapes, respectively.

| Item | | NO.1 | NO.2 | NO.3 | NO.4 | NO.5 | NO.6 | NO.7 | NO.8 | NO.9 | NO.10 | NO.11 | NO.12 | NO.13 | NO.14 | NO.15 |
| --- | --- | --- | --- | --- | --- | --- | --- | --- | --- | --- | --- | --- | --- | --- | --- | --- |
| FR | AGV | 4,103 | 1,295 | 5,135 | 1,136 | 2,078 | 1,456 | 1,589 | 1,671 | 1,205 | 4,981 | 8,511 | 5,335 | 5,994 | 4,123 | 1,140 |
|  | AIA | 1,174 | 338 | 1,220 | 251 | 560 | 341 | 471 | 440 | 304 | 634 | 1,385 | 1,132 | 1,083 | 1,015 | 330 |
| SL | AGV | 112,664 | 59,760 | 104,071 | 160,661 | 91,600 | 36,940 | 64,195 | 13,224 | 2,285 | 45,568 | 125,443 | 13,258 | 23,437 | 70,107 | 95,147 |
|  | AIA | 6,898 | 4,668 | 9,367 | 7,057 | 6,212 | 3,363 | 6,640 | 1,146 | 540 | 6,317 | 7,045 | 3,039 | 3,277 | 7,845 | 7,339 |
| MO | AGV | 152,851 | 57,944 | 73,375 | 42,500 | 127,206 | 60,994 | 122,914 | 80,139 | 27,849 | 183,937 | 100,492 | 105,067 | 157,747 | 103,448 | 162,337 |
|  | AIA | 6,935 | 5,626 | 6,502 | 6,319 | 9,255 | 7,703 | 11,500 | 8,650 | 4,059 | 9,988 | 6,961 | 11,448 | 6,996 | 5,558 | 6,662 |
| SR | AGV | 237,342 | 466,254 | 412,736 | 184,395 | 499,795 | 719,501 | 138,842 | 230,229 | 714,251 | 1,097,902 | 203,224 | 243,831 | 594,585 | 749,751 | 911,293 |
|  | AIA | 9,588 | 13,240 | 18,130 | 15,006 | 16,155 | 14,415 | 12,374 | 6,737 | 15,290 | 16,915 | 11,320 | 16,062 | 17,319 | 15,863 | 16,672 |
